# Applications of artificial neural networks in health care organizational decision-making: A scoping review

**DOI:** 10.1371/journal.pone.0212356

**Published:** 2019-02-19

**Authors:** Nida Shahid, Tim Rappon, Whitney Berta

**Affiliations:** 1 Institute of Health Policy, Management and Evaluation, University of Toronto, Toronto, Canada; 2 Toronto Health Economics and Technology Assessment (THETA) Collaborative, University Health Network, Toronto, Canada; The University of Warwick, UNITED KINGDOM

## Abstract

Health care organizations are leveraging machine-learning techniques, such as artificial neural networks (ANN), to improve delivery of care at a reduced cost. Applications of ANN to diagnosis are well-known; however, ANN are increasingly used to inform health care management decisions. We provide a seminal review of the applications of ANN to health care organizational decision-making. We screened 3,397 articles from six databases with coverage of Health Administration, Computer Science and Business Administration. We extracted study characteristics, aim, methodology and context (including level of analysis) from 80 articles meeting inclusion criteria. Articles were published from 1997–2018 and originated from 24 countries, with a plurality of papers (26 articles) published by authors from the United States. Types of ANN used included ANN (36 articles), feed-forward networks (25 articles), or hybrid models (23 articles); reported accuracy varied from 50% to 100%. The majority of ANN informed decision-making at the micro level (61 articles), between patients and health care providers. Fewer ANN were deployed for intra-organizational (meso- level, 29 articles) and system, policy or inter-organizational (macro- level, 10 articles) decision-making. Our review identifies key characteristics and drivers for market uptake of ANN for health care organizational decision-making to guide further adoption of this technique.

## Introduction

As health care systems in developed countries transform towards a value based, patient-centered model of care delivery, we face new complexities relating to improving the structure and management of health care delivery; for example, improving integration of processes in care delivery for patient-centered chronic disease management [1]. Artificial intelligence lies at the nexus of new technologies with the potential to deliver health care that is cost-effective and appropriate care in real-time, manage effective and efficient communication among multidisciplinary stakeholders, and address non-traditional care settings, the evolving heathcare workplace and workforce, and the advent of new and disparate health information systems. With the rapid uptake of artificial intelligence to make increasingly complex decisions across different industries, there are a multitude of solutions capable of addressing these health care management challenges; however, there is a paucity of guidance on selecting appropriate methods tailored to the health care industry[2].

Global health care expenditure is expected to reach $8.7 trillion by 2020, driven by aging populations growing in size and disease complexity, advancements made in medical treatments, rising labour costs and the market expansion of the health care industry. Many health systems are reported to struggle with updating aging infrastructure and legacy technologies with already limited capital resources. In an effort toward moving to value-based care, decision-makers are reported to be strategically shifting the focus to understanding and better alignment of financial incentives for health care providers in order to bear financial risk; population health management including analyses of trends in health, quality and cost; and adoption of innovative delivery models for improved processes and coordination of care.

Health care organizations are required to be increasingly strategic in their management due to a variety of system interdependences such as emerging environmental demands and competing priorities, that can complicate decision-making process [3]. According to economy theory, most organizations are risk-aversive [4] and decision-makers in health care can face issues related to culture, technology and risk when making high-risk decisions without the certainty of high-return [4, 5]. Patient care and operations management requires the interaction of multiple stakeholders, for example clinicians, front-line/middle managers, senior level executives to make decisions on a clinical (e.g. diagnosis, treatment and therapy, medication prescription and administration), and non-clinical (e.g. budget, resource allocation, technology acquisition, service additions/reductions, strategic planning) [6].

A white paper published by IBM suggests that with increasing capture and digitization of health care data (e.g. electronic medical records and DNA sequences), health care organizations are taking advantage of analyzing large sets of routinely collected digital information in order to improve service and reduce costs [7]. Reported examples include analyzing clinical, financial and operational data to answer questions related to effectiveness of programs, making predictions regarding at-risk patients. The global market for health care predictive analytics is projected was valued at USD 1.48 billion in 2015 and expected to grow at a rate of 29.3% (compound annual growth rate) by 2025 [8]. Similarly, global revenue of $811 million is expected to increase 40% (Compound Annual Growth Rate) by 2021 due the artificial intelligence (AI) market for health care applications. A subfield of AI, machine learning-as-a-service-market (MLaaS), is expected to reach $5.4 billion by 2022, with the health care sector as a notable key driver [9].

A recent survey of AI applications in health care reported uses in major disease areas such as cancer or cardiology and artificial neural networks (ANN) as a common machine learning technique [10]. Applications of ANN in health care include clinical diagnosis, prediction of cancer, speech recognition, prediction of length of stay [11], image analysis and interpretation [12] (e.g. automated electrocardiographic (ECG) interpretation used to diagnose myocardial infarction [13]), and drug development[12]. Non-clinical applications have included improvement of health care organizational management [14], prediction of key indicators such as cost or facility utilization [15]. ANN has been used as part of decision support models to provide health care providers and the health care system with cost-effective solutions to time and resource management [16].

### Rationale

Despite its many applications and, more recently, its prominence [17], there is a lack of coherence regarding ANN’s applications and potential to inform decision making at different levels in health care organizations. This review is motivated by a need for a broad understanding the various applications of ANN in health care and aids researchers interested in bridging the disciplines of organizational behaviour and computer science. Considering the sheer abundance in reported use and complexity of the area, it can be challenging to remain abreast of the new advancements and trends in applications of ANN [18]. Adopters of ANN or researchers new to the field of AI may find the scope and esoteric terminology of neural computing particularly challenging [18]. Literature suggests that current reviews on applications of ANN are limited in scope and generally focus on a specific disease [19] or a particular type of neural network [20], or they are too broad (i.e. data mining or AI techniques that can include ANN but do not offer insights specific to ANN) [10]. The overarching goal of this scoping review is to provide a much-needed comprehensive review of the various applications of ANN in health care organizational decision-making at the micro-, meso-, and macro-levels. The levels pertain to decisions made on the (micro) level of individual patients, or on a (meso) group level (e.g. departmental or organizational level) where patient preference may be important but not essential; and on a wider (macro) level by large groups or public organizations related to allocation or utilization of resources where decisions are based on public interest and reflective of society as a whole [21]. By means of this review, we will identify the nature and extent of relevant literature and describe methodologies and context used.

### Overview

According to an overview by Kononenko (2001), as a sub-field of AI, machine learning provides indispensable tools for intelligent data analysis. Three major branches of machine learning have emerged since electronic computers came in to use during the 1950s and 1960s: statistical methods, symbolic learning and neural networks [22]. ANN have been successfully used to solve highly complex problems within the physical sciences and as of late by scholars in organizational research as digital tools enabling faster processes of data collection and processing [23]. As practical and flexible modelling tools, ANN have an ability to generalize pattern information to new data, tolerate noisy inputs, and produce reliable and reasonable estimates [23]. ANN belong to a wide class of flexible nonlinear regression and discriminant models, data reduction models, and nonlinear dynamical systems [24]. ANN are similar to statistical techniques including generalized linear models, nonparametric regression and discriminant analysis, or cluster analysis [24]. As a statistical model, it’s general composition is one made of simple, interconnected processing elements that are configured through iterative exposure to sample data [23]. Its application is particularly valuable under one or more of several conditions: when sample data show complex interaction effects or do not meet parametric assumptions, when the relationship between independent and dependent variables is not strong, when there is a large unexplained variance in information, or in situations where the theoretical basis of prediction is poorly understood [23]. ANN architectures are commonly classified as feed-forward neural networks (e.g. single-layer perceptron, multi-layer perceptron, radial basis function networks) or feed-back, or otherwise referred to as recurrent neural networks (e.g. Competitive networks, Kohonen’s self-organizing maps, Hopfield networks) [25].

## Artificial neural networks

Originally developed as mathematical theories of the information-processing activity of biological nerve cells, the structural elements used to describe an ANN are conceptually analogous to those used in neuroscience, despite it belonging to a class of statistical procedures [23].

### Basics

ANN can have single or multiple layers [23], and consist of processing units (nodes or neurons) that are interconnected by a set of adjustable weights that allows signals to travel through the network in parallel and consecutively[13, 26]. Generally ANN can be divided in to three layers of neurons: input (receives information), hidden (responsible for extracting patterns, perform most of internal processing), and output (produces and presents final network outputs) [27].

A review by Agatonovic-Kustrin & Beresford (2000) describes neural computation to be powered from the connection of its neurons and that each neuron has a weighted input, transfer function and a single output. The authors state that the neuron is activated by the weighed sum of inputs it receives and the activation signal passes through a transfer function to produce a single output. The transfer functions, the learning rule and the architecture determine the overall behaviour of the neural network [26].

### Architecture

**Fig 1:**
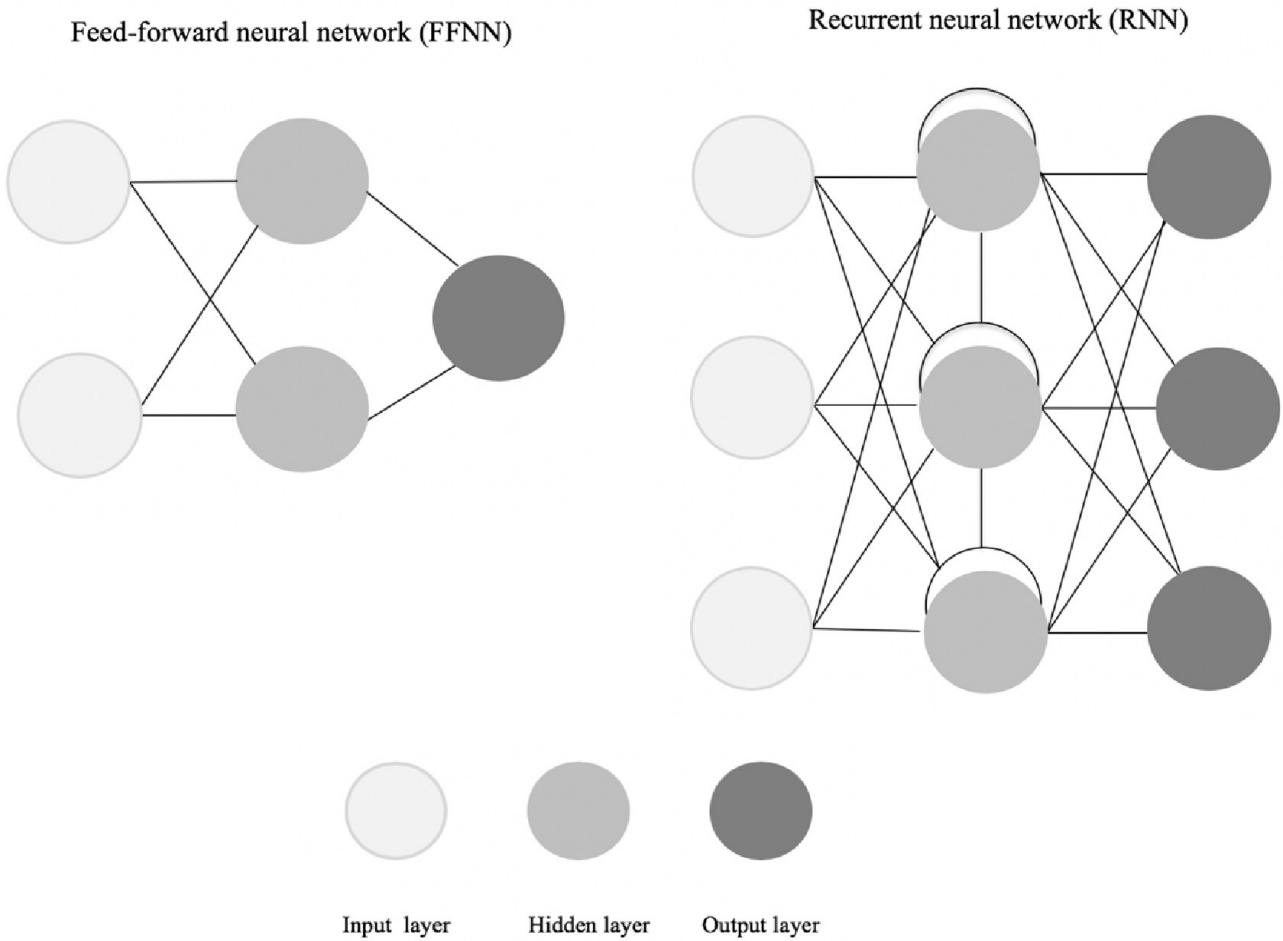
Conceptual model of a feed-forward and recurrent neural network.

Sharma & Chopra (2013) describe the two most common types of neural networks applied in management sciences to be the feed-forward and recurrent neural networks (Fig 1) in comparison with feed-forward networks common to medical applications [28, 29]. A feed-forward network can be single-layered (e.g. Perceptron, ADALINE) or multi-layered (e.g. Multilayer Perceptron, Radial Basis Function) [27, 30]. Sharma & Chopra (2013) describe information flow in feed-forward networks to be unidirectional from input layer, through hidden layers to the output layer, without any feedback. Whereas, a recurrent or feedback network involves dynamic information processing having at least one feedback loop, using outputs as feedback inputs (e.g. Hopfield) [27, 30]. Fig 1 illustrates the two types of networks with three layers (input, hidden and output).

### Learning

In an overview of basic concepts, Agatonovic-Kustrin & Beresford (2000) describe ANN gather knowledge by detecting patterns and relationships in data and “learn” through experience. The authors state an artificial neural network learns by optimizing its inner unit connections in order to minimize errors in the predictions that it makes and to reach a desired level of accuracy. New information can be inputted into the model once the model has been trained and tested [26]. Also referred to as the generalized delta rule, backpropagation refers to how an ANN is trained or ‘learns’ based on data. It uses an iterative process involving six steps: (i) single case data is passed to input later, output is passed to the hidden layer and multiplied by the first set of connection weights; (ii) incoming signals are summed, transformed to output and passed to second connection weight matrix; (iii) incoming signals are summed, transformed and network output is produced; (iv) output value is subtracted from known value for that case, error term is passed backward through network; (v) connection weights are adjusted in proportion to their error contribution; (vi) modified connection weights saved for next cycle, next case input set queued for next cycle [23]. Sharma & Chopra (2013) broadly classify training or ‘learning’ methods in ANN into three types: supervised, unsupervised and reinforced learning. In supervised learning, every input pattern used to train the network is associated with an output pattern. The error in computed and desired outputs can be used to improve model performance. In unsupervised learning, the network learns without knowledge of desired output and by discovering and adapting to features of the input patterns. In reinforcement learning, the network is provided with feedback on if computation performance without presenting the desired output [30].

## Artificial neural networks and regression models

Neural networks are similar to linear regression models in their nature and use. They are comprised of input (independent or predictor variable) and output (dependent or outcome variable) nodes, use connection weights (regression coefficients), bias weight (intercept parameters) and cross-entropy (maximum likelihood estimation) to learn or train (parameter estimation) a model [31]. ANN learn to perform tasks by using inductive learning algorithms requiring massive data sets [18]. A working paper on the use of ANN in decision support systems states that the structure, quality and quantity of data used is critical for the learning process and that the chosen attributes must be complete, relevant, measurable and independent[18]. The authors further observe that in business applications, external data sources (e.g. industry and trade databases) are typically used to supplement internal data sources.

### Classification and prediction modelling

In the book entitled ‘Data Mining: Concepts and Techniques', classification is defined as the process of finding a model that describes and distinguishes data classes or concepts based on analysis of a set of training data [32]. The authors write that models called classifiers predict *categorical* class labels and can be used to predict the class label of objects for which the class label is unknown. Furthermore, the process is described to consist of a learning step (when a classification model is constructed) and a classification step (when a model is used to predict class labels for a given data). Methods include naïve Bayesian classification, support vector machines, and *k*-nearest-neighbour classification [32]. Han et al. (2012) suggest that applications can broadly include fraud detection, target marketing, performance prediction, manufacturing and medical diagnosis.

The available data is divided into two sets for cross-validation: a training set used to develop a model and a test set, used to evaluate the model’s performance [33, 34]. Appropriate data splitting is a technique commonly used in machine learning in order to minimize poor generalization (also referred to as over-training or over-fitting) of models [34]. Using more training data improves the classification model, whereas using more test data contributes to estimating error accurately [35]. Although a 70:30 ratio can typically be used for training/testing size [36], various statistical sampling techniques ranging from simple (e.g. simple random sampling, trial-and-error) to more deterministic (e.g. CADEX, DUPLEX) can be used to split the data depending on the goals and complexity of the problem [34].

Han and colleagues (2012) write that where classification predicts categorical labels, regression is used to predict missing or unavailable *numerical* data values (rather than discrete class labels). The authors describe regression analysis as a statistical methodology often used for numeric prediction and encompasses identification of distribution trends based on available data. An example of numeric prediction is when a model is constructed to predict a continuous-valued function or ordered value (as opposed to a class label). Such a model is called a predictor model and typically uses regression analysis [32].

ANN can be used to perform nonlinear statistical modeling and provide new alternatives to logistic regression, the most commonly used method for developing predictive models for dichotomous outcomes in medicine [31]. Users require less formal statistical training and the networks are able to detect complex non-linear relationships and interactions between dependent and independent variables. ANN can combine and incorporate literature-based and experimental data to solve problems [26]. Other advantages of ANN, relative to traditional predictive modeling techniques, include fast and simple operation due to compact representation of knowledge (e.g., weight and threshold value matrices), the ability to operate with noisy or missing information and generalize to similar unseen data, the ability to learn inductively from training data and process non-linear functionality critical to dealing with real-word data [37].

Although ANN do not require knowledge of data source, they require large training sets due to the numerous estimated weights involved in computation [26]. They may require lengthy training times and the use of random weight initializations may lead to different solutions [37]. Despite successful applications, ANN remain problematic in that they offer us little or no insight into the process(es) by which they learn or the totality of the knowledge embedded in them [38]. Several limitations of ANN are identified in the literature: they are limited in their ability to explicitly identify possible causal relationships, they are challenging to use in the field, they are prone to over fitting, model development is empirical potentially requiring several attempts to develop an acceptable model [37], and there are methodological issues related to model development [31]. In comparing advantages and disadvantages of using ANN to predict medical outcomes, Tu (1996) suggests that logistic regression models can be disseminated to a wider audience, whereas ANN models are less transparent and therefore can be more difficult to communicate and use. Even if published and made available, the connection weight matrices used in ANN for training a data set may be large and difficult to interpret for others to make use of, whereas logistic regression coefficients can be published for any end user to be able to calculate [31].

## Methods

The Arksey & O’Malley framework (2005) was adopted to identify the (i) research question, (ii) relevant studies, (iii) select studies, (iv) chart the data and (v), collate, summarize and present findings.

### Search strategy

Due to the cross-disciplinary nature of our query, the search strategy was designed to identify literature from multiple databases according to the key disciplines of Health Administration (Medline and Embase), Computer Science (ACM Digital Library and Advanced Technologies & Aerospace Database), and Business and Management (ABI/Inform Global and JSTOR). The selection of the three disciplines reflects the core concepts embedded in our research question: ‘what are the different applications of ANN (Computer Science) in health care organizational decision-making (Health Administration and Business Management)?’

In consultation with a librarian, a comprehensive search syntax was built on the concepts of ‘artificial neural networks’ applied in ‘health care organizational decision-making’ and tailored for each database for optimum results. The final search syntax was based on search terms refined through an iterative process involving examination of a preliminary set of results to ensure relevance (S1 Appendix). The search strategy was limited to peer-reviewed publications in English without limitation to the year of publication up until the time of our search (January 2018). Our background search did not identify seminal paper(s) published or advancements related to our research question, thereby justifying the rationale for not limiting the search to a specicic start date.

### Data collection

Screening of articles occurred in two stages. Identified articles were de-duplicated and imported to EndNote as a reference manager and to Covidence, a web-based platform, for screening. The screening inclusion and exclusion criteria were built iteratively via consensus (NS, TR and WB) (Table 1). Titles and abstracts were first screened to include articles with keywords related to and/or in explicit reference to artificial neural networks. Articles were excluded if there was no explicit reference to artificial neural networks; the application was not in the health care domain or context of health care organizational decision-making, or was not a publication that was peer-reviewed (e.g. grey literature e.g. conference abstracts and papers, book reviews, newspaper or magazine articles, teaching courses). Table 1 lists the criteria used to screen, include or exclude articles in the review.

**Table 1:** Screening inclusion, exclusion criteria.

	Inclusion criteria	Exclusion criteria
**Titles and abstracts**	Explicit reference to keywords: neural network; artificial neural network; ANNs;	Does not make explicit reference to artificial neural networks within the context of healthcare or medicine
	Must make reference to ANN if any type of artificial intelligence or machine learning techniques used, (e.g. Fuzzy logic, Bayesian statistics and Self-Organizing Maps, back-propagation; prediction model; unsupervised learning)	
**Publication Type**	Peer-reviewed empirical or theoretical work (e.g. Journal articles, reports)	Not based on empirical or theoretical work (e.g. book reviews, newspaper article, course material); conference papers and abstracts
**Setting or Context**	Application in domain of Healthcare and/or Medicine	Application was not directly related to healthcare organizational decision making (e.g. speech recognition)

Subsequently, a full-text review of articles that met the initial screening criteria was conducted on basis of relevance and availability of information for data extraction. In addition to independent review and extraction of articles, two coders (NS and TR) extracted data from a subset of articles for consensus, minimization of error, and clarity between reviewers regarding the choice of data selected for extraction. Information related to study characteristics, aim, methodology (application, taxonomy, accuracy) and context including organizational level of analysis (micro-, meso- and macro-) was collected and entered into Microsoft Excel for categorization and descriptive analysis. Applications of ANN to make decisions directly between providers and patients was categorized as ‘micro’, any decisions made by a larger group and not directly related to a patient was categorized as ‘meso’, and decisions beyond an organizational group (i.e. across different institutions, a system or countries) was categorized as ‘macro’ level of decision-making.

## Results

**Fig 2:**
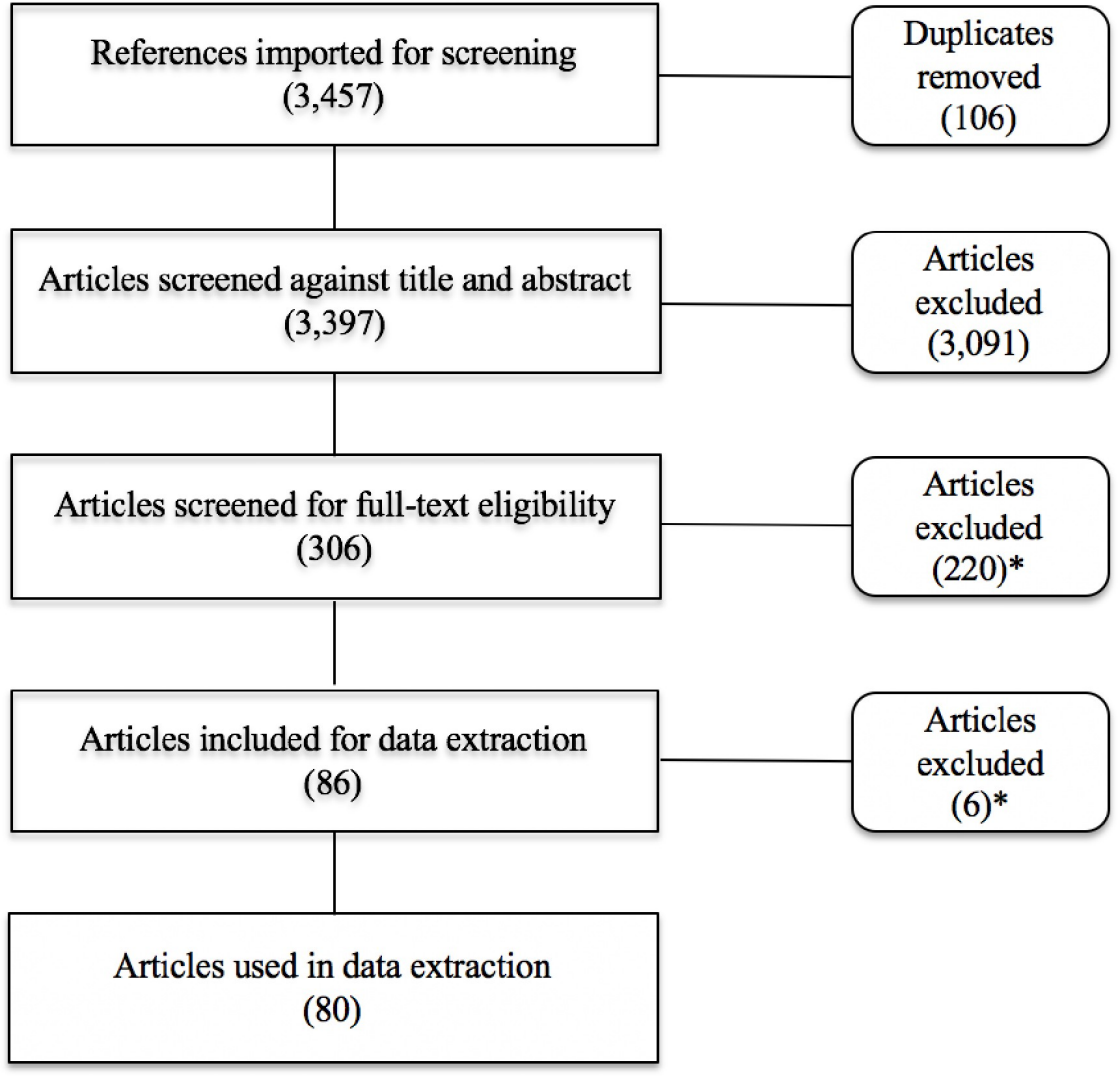
Review process overview. *Articles excluded for the following reasons: Not ANN or suitable synonym (n = 93), use of ANN unrelated to healthcare organizational decision-making (n = 70), based on iterated exclusion criteria (n = 45), not based on empirical or theoretical research (n = 9), could not access full-text (n = 9).

Overall, 3,457 articles were imported for screening, out of which (after removal of duplicates) 3,397 were screened for titles and abstracts to give a total of 306 articles used for full-text review (Fig 2). Articles were excluded from data collection for reasons such as: there being no explicit reference to ANN being used (91 articles), the application of ANN was not in the context of health care organizational decision-making (68 articles), on basis of study exclusion criteria (53 articles) or the articles were irretrievable (8). In total, 80 articles were used for data collection. Fig 2 illustrates the overall review process including number of articles excluded at each stage.

### Study characteristics

**Fig 3:**
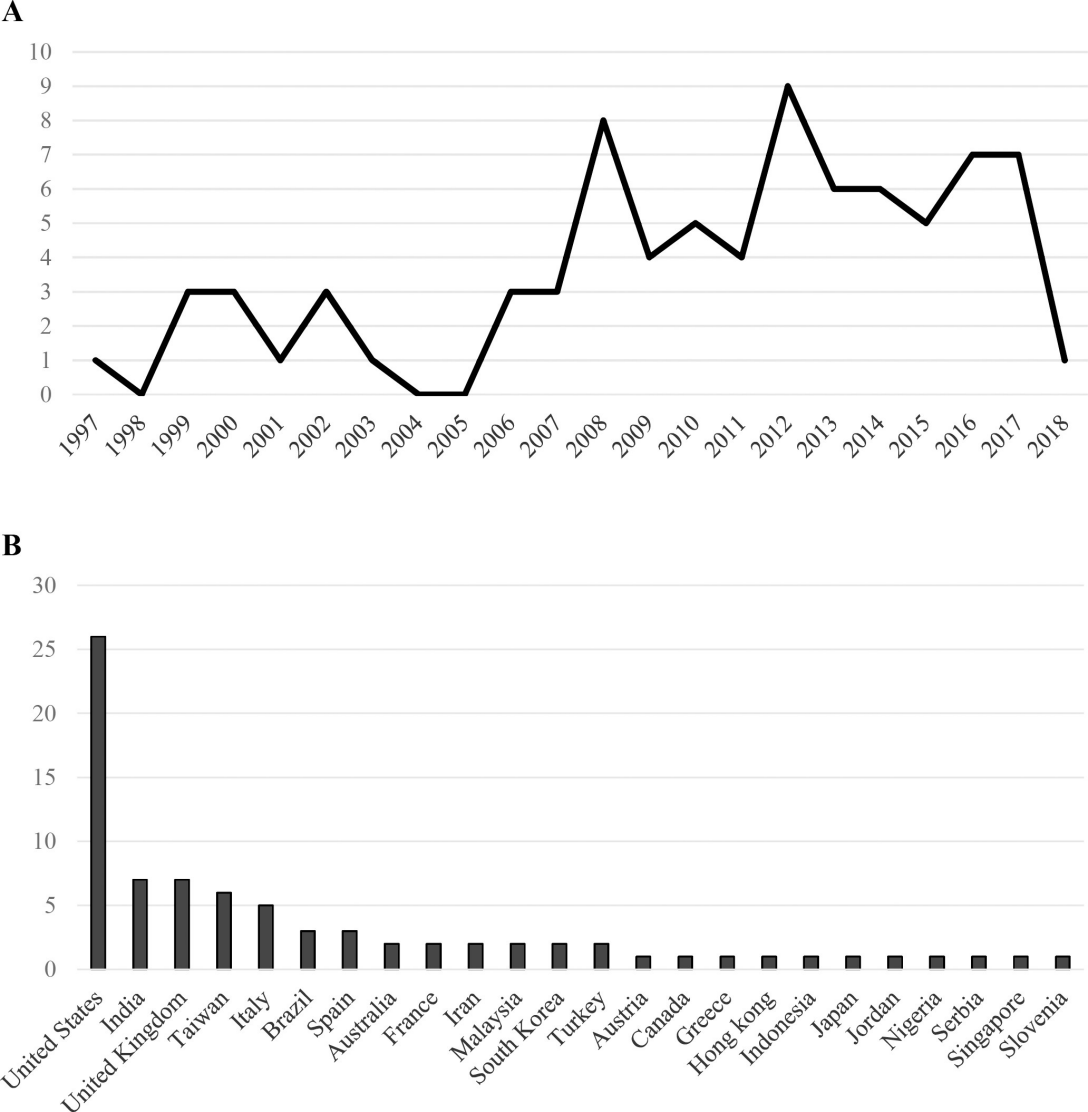
Article characteristics. (A) Number of articles by publication year. (B) Number of articles by country.

Publication dates ranged from 1997 to 2018 with the number of studies fluctuating each year (Fig 3A). Studies were published across 24 countries with the majority of first authors from the United States (26), the United Kingdom and India (7), Taiwan (6) and Italy (5) (Fig 3B). Fig 3A and 3B illustrate the number of articles published over the years and across varying countries.

### Aim and methodology

Main topics or area of interest based on the article’s overall purpose included Organizational Behaviour (18%), Cardiovascular (14%), Infectious Disease and Telemedicine (7%) (Table 2). Topics categorized under ‘Organizational Behaviour’ include: behaviour and perspectives, crisis or risk management, clinical and non-clinical decision-making, and resource management (S2 Appendix). Table 2 lists the main topic areas of articles reviewed.

**Table 2:** Study areas identified in the review.

Study Area	Number of Articles
Organizational Behaviour	18
Other*	15
Cardiovascular	14
Infectious Disease	7
Telemedicine	7
Finance	5
Trauma	5
Medical Imaging	4
Diabetes	4
Surgery	4
Information Systems	4

*Sub-categories of ‘Other’ articles include: elderly studies, renal disease, medical diagnosis, data mining, pharmacology, fall detection, disorders (epilepsy or autism).

**Fig 4:**
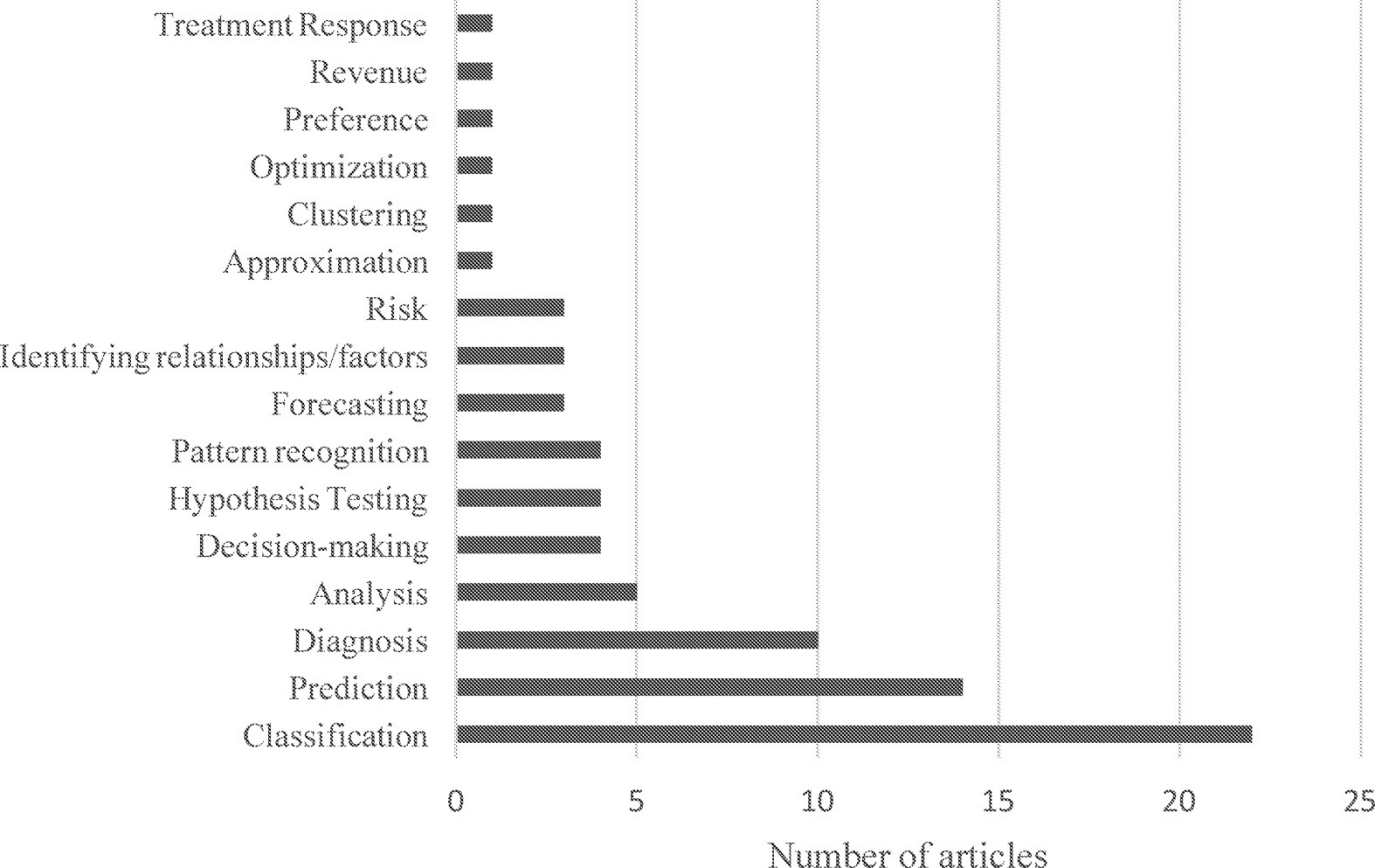
Types of applications of artificial neural networks identified in the review.

Applications of ANN were mainly found to be classification (22), prediction (14), and diagnosis (10) (Fig 4). Examples of applications include classification of data in medical databases (i.e. organizing or distinguishing data by relevant categories or concepts) [39], using a hybrid learning approach for automatic tissue recognition in wound images for accurate wound evaluations [40], and comparison of soft-computing techniques for diagnosis of heart conditions by processing digitally recorded heart sound signals to extract time and frequency features related to normal and abnormal heart conditions [41]. Applications for prediction included developing a risk advisor model to predict the chances of diabetes complication according to changes in risk factors [42], identifying the optimal subset of attributes from a given set of attributes for diagnosis of heart disease [43], modelling daily patient arrivals in the Emergency Department [44]. ANN was applied for diagnosis of disease based on age, sex, body mass index, average blood pressure and blood serum measurements [45], comparing predictive accuracies of different types of ANN and statistical models for diagnosis of coronary artery disease [46], diagnosis and risk group assignment for pulmonary tuberculosis among hospitalized patients [47], and non-invasive diagnosis of early risk in dengue patients [48]. Other examples include exploring the potential use of mobile phones as a health promotional tool by tracking daily exercise activities of people and using ANN to estimate a user’s movement[49], or using ANN to identify factors related to treatment and outcomes potentially impacting patient length of stay[50]. In addition to S2 Appendix, Fig 4 illustrates the various applications of ANN identified in the literature review.

With respect to nomenclature or taxonomy, authors mostly reported using artificial neural networks (36 articles), feed-forward networks (25 articles), a hybrid model (23 articles), recurrent feedback networks (6 articles) or other (3 articles) (S2 Appendix). Various types of data (e.g. patients, cases, images, and signals) and sample sizes were used. Training/testing sets were in ratios of 50:50, 70:30 or 90:10 and the reported accuracy ranged between 50% and 100%.

### Context and key findings

ANN was primarily applied to organizational decision-making at a micro-level (61 articles) between patients and health care providers in addition to meso-, macro-levels out of which 48 articles referenced to micro-level decision-making only; with 29 articles referencing meso-level applications between patients, health care providers, hospital managers and decision-makers, out of which 10 referenced meso- only. A small portion (10) of studies applied ANN at a macro level of decision-making mainly between policy and decision-makers across multiple facilities or health care systems, out of which 2 referenced macro- only. Micro-level applications of ANN include diagnosis of pulmonary tuberculosis among hospitalized patients by health care providers using models developed for classification and risk group assignment [47], classify Crohn’s Disease medical images [51], analyse recorded ECG signals to trigger an alarm for patients and allow collection and transmission of patient information to health care providers[52]. Meso-level applications include decision-making among managers involving classification of cost [53], developing a forecasting model to support health care management decision-making[54], among patients, providers, and hospital managers in order to evaluate the effect of hospital employee motivation on patient satisfaction [55], and predicting the adoption of radio frequency identification (RFID) technology adoption in clinical setting [56]. Macro-level applications of ANN include risk-adjustment models for policy-makers of Taiwan’s National Health Insurance program [57], a global comparison of the perception of corruption in the health care sector [58], model revenue generation for decision-makers to determine best indicators of revenue generation in not-for-profit foundations supporting hospitals of varying sizes [59].

Authors reported neural networks reduced computation time in comparison to conventional planning algorithms [60] thereby enabling users to access model output faster in real-time, outperforming linear regression models in prediction [44, 56, 61–63] and support vector machines in classification [64, 65]. Limitations centered around the use of small data sets [42, 53, 66–72], limiting data set to continuous variables [69], inability to examine causal relationships [56] or have the network explain weights applied, appropriateness of decision-making [71, 73, 74], difficulty in implementation or understanding of the output [75]. ANN were cautioned to be used as a proof of concept rather than a successful prediction model [66].

## Discussion

This review provides a comprehensive review of the various applications of artificial neural networks in health care organizational decision-making. To our knowledge, this is the first attempt to comprehensively describe the use of ANN in health care, from the time of its origins to current day use, on all levels of organizational decision-making.

Prior efforts have concentrated on a specific domain or aspect of health care and/or limited study findings to a period of time. A systematic review on the use of ANN as decision-making tools in the field of cancer reported trends from 1994–2003 in clinical diagnosis, prognosis and therapeutic guidance for cancer from1994 to 2003, and suggested the need for rigorous methodologies in using neural networks [19]. Another review reported various applications in areas of accounting and finance, health and medicine, engineering and marketing, however focused the review on feed-forward neural networks and statistical techniques used in prediction and classification problems [20]. Outside of medicine and health care, Wong et al. conducted literature reviews of ANN used in business (from 1988–1995) [76] and finance (1990–1996) [77], at that time describing the promise of neural networks for increasing integration with other existing or developing technologies [76, 77]. Data mining is the mathematical core of a larger process of knowledge discovery from databases otherwise referred to as the ‘KDD process [78]. The main activities involved in the KDD process include (i) integration and cleaning, (ii) selection and transformation, (iii) data mining and (iv) evaluation and interpretation. Data mining pertains to extraction of significant patterns and knowledge discovery and employs inferring algorithms, such as ANN, to pre-processed data to complete data mining tasks such as classification and cluster analysis [79]. Data mining and machine learning have produced practical applications in areas of analysing medical outcomes, detecting credit card fraud, predicting customer purchase behaviour or predicting personal interests from internet use [80]. Although limited in scope to the field of infertility, Durairaj & Ranjani (2013) conducted a comparative study of data mining techniques including ANN, suggesting the promise of combining more than one data mining technique for diagnosing or predicting disease [81].

Due to the primitive nature of computer technology mid-20^th^ Century, most of the research in machine learning was theoretical or based on construction of special purpose systems [18]. We found that application of ANN in health care decision-making began in the late 90’s with fluctuating use over the years. A number of breakthroughs in the field of computer science and AI bring insight to reported publication patterns [82]. ANN gained prominence with the publication of a few seminal works including the publication of the backpropagation learning rule for multilayered feed-forward neural networks [22]. In 1986, backpropagation was proven as a general purpose and simple procedure, powerful enough for a multi-layered neural network to use and construct appropriate internal representations based on incoming data [83]. A few years later, the ability of neural networks to learn any type of function was demonstrated [84], suggesting capabilities of neural networks as universal approximators [85]. During the 90’s, most of the research was largely experimental and the need for use of ANN as a widely-used computer paradigm remained warranted [18].

With the digitization of health care [86], hospitals are increasingly able to collect large amounts of data managed across large information systems [22]. With its ability to process large datasets, machine learning technology is well-suited for analysing medical data and providing effective algorithms [22]. Considering the prevalent use of medical information systems and medical databases, ANN have found useful applications in biomedical areas in diagnosis and disease monitoring [87].

Although the backpropagation learning rule enabled the use of neural networks in many hard medical diagnostic tasks, they have been typically used as black box classifiers lacking the transparency of generating knowledge as well as the ability to explain decision-making [22]. The lack of transparency or interpretability of neural networks continues to be an important problem since health care providers are often unwilling to accept machine recommendations without clarity regarding the underlying rationale [88]. Prior to 2006, application of neural networks included processing of biomedical signals, for example image and speech processing [89, 90], clinical diagnosis, image analysis and interpretation, and drug development [87]. In 2006, a critical paper described the ability of a neural network to learn faster [91]. Six years later, the largest deep neural network to date (i.e. depth pertaining to layers of the network), was trained to classify 1.2 million images in record-breaking time as part of the ImageNet Large Scale Visual Recognition Challenge [92].

The most successful applications of ANN are found in extremely complex medical situations [13]. We found ANN to be mainly used for classification, prediction and clinical diagnosis in areas of cardiovascular, telemedicine and organizational behaviour. Use of ANN applies to four general areas of cardiovascular medicine: diagnosis and treatment of coronary artery disease, general interpretation of electrocardiography, cardiac image analysis and cardiovascular drug dosing [93]. Telemedicine offers health care providers elaborate solutions for remote monitoring designed to prevent, diagnose, manage disease and treatment [94] and can include machine learning techniques to predict clinical parameters such as blood pressure [95]. Preliminary diagnosis of high-risk patients (for disease or attributes) using neural networks provide hospital administrators with a cost-effective tool in time and resource management [16].

Neural networks have been used effectively as a tool in complex decision-making in strategic management, specifically in strategic planning and performance, assessing decision-making [96]. In health care, neural network models have been successfully used to predict quality determinants (responsiveness, security, efficiency) influencing adoption of e-government services [97]. With its ability to discover hidden knowledge and values, scholars have suggested using ANN to improve care performance and facilitate the adoption of ‘Lean thinking’ or value-based decision making in health care [87]. An example of ANN facilitating Lean thinking adoption in health care contexts is its application to describe ‘information flow’ among cancer patients by modeling the relationship between quality of life evaluations made by patients, pharmacists and nurses [87]. ‘Flow’ is a key concept in a Lean System and ‘information flow’ is an essential improvement target to the successful operation of a health care system using a Lean approach [87]. Key success factors or differentiators that define effective machine learning technology in health care include access to extensive data sources, ease of implementation, interpretability and buy-in as well as conformance with privacy standards [9]. Support vector machines are used to model high-dimensional data and are considered state-of-the-art solutions to problems otherwise not amenable to traditional statistical analysis. Despite its analytic capabilities, wide-scale adoption remains a challenge, mainly due to methodological complexities and scalability challenges [98]. For example, a systematic review of deep learning models using electronic health record data recently identified challenges related to the temporality (e.g. hidden relationships among clinical variables occurring at short and long term events) and irregularity of information used which can reduce model performance if not handled appropriately [88]. Poor interpretability remains a signicant challenge with implementing ANN in health care [90]. Zhang et al (2018) report that in comparison to linear models, ANN are not only difficult to interpret but the identification of predictors (input features) important for the model also seem to be a challenge [99]. Fisher et al (2016) developed an ANN based monitoring method evaluating Parkinson’s disease motor symptoms and reported signiciant challenges with detecting disease states due to the inherent subjectivity underlying the interpretation of disease state descriptors (i.e. the degree of motor symptoms experienced by each patient would likely vary) [100]. Despite the evident progress in certain areas (e.g. knowledge and temporal representation, machine learning), the adoption of key standards required for integration and knowledge sharing (e.g. controlled terminologies, semantic structuring, standards representing clinical decision logic) has been slow [101] Patel et al. (2009) suggest barriers to progress are related to political, fiscal or cultural reasons and not purely technical. A national study on the implementation of Health Information Technology (HIT) in the United States reported a poor understanding of IT staff, informaticians, health information managers and others playing a significant role in implementation of HIT in health care [102] Barriers to adoption of HIT include mismatch of return on investment, challenges to workflow in clinical settings, lack of standards and interoperability, and concerns about privacy and confidentiality [102].

We found that researchers often adopted a hybrid approach when using neural networks. Hybrid approaches (e.g. combining two or more techniques/soft-computing paradigms) are effective in reducing challenges with neural networks when introducing new items to the system or having insufficient data [103]. ANN learn (supervised, unsupervised or reinforcement) based on the iterative adjustment of connection weights using optimization algorithms such as the backpropagation rule. Challenges related to such algorithms include the necessity of a previously defined architecture for the model, sensitivity to the initial conditions used in training [104]. A hybrid model of an ANN and decision tree classifier has been used to predict university admissions using data related to student academic merits, background and university admission criteria. Reported advantages of using a hybrid model included higher prediction accuracy rates (error rate of <2%), flexibility and faster performance (0.1 second) in comparison with a model using neural networks only (20 minutes learning time). Another advantage reported was improved generalizability, e.g. ability to understand rules extracted that can be later coded into another type of system [105] Literature suggests extensive use of ANN in business applications in particular areas related to financial distress and bankruptcy problems, stock price forecasting and decision support [106] Hybrid networks have also been developed in business applications to improve performance of standard models [106]. The integration of ANN with secondary AI and meta-heuristic methods such as fuzzy logic, genetic, bee colony algorithms, or artificial immune systems have been proposed to reduce or eliminate challenges related to ANN (e.g. selection of network topology, initial weights, choice of control parameters) [106]. Applications of hybrid intelligent systems include robotics, medical diagnosis, speech/natural language understanding, monitoring of manufacturing processes.

Our findings suggest a possible correlation between advancements made in the field of ANN and publication rates related to the application of ANN in health care organizational decision-making. Despite the variety of study contexts and applications, ANN continues to be mainly used for classification, prediction and diagnosis. As suggested by the literature, the most commonly used taxonomy of ANN found was the feed-forward neural network. However, our study showed a significant use of hybrid models. ANN’s application to facilitate more micro- and meso-level decision-making compared to macro-level may be explained by the type and volume of data required and available to build an effective model.

## Strengths and limitations

A primary strength of this review is its comprehensive scope and search strategy involving multiple databases. Variables selected for data collection were based on bodies of work with similar inquiry and well aligned with the methods of a scoping review. The complex nature of artificial neural networks required a fundamental understanding for the authors who were otherwise novice to the field. Studies included in this review did not always use standardized reporting measures and may include publications of lower quality.

## Implications

### Practical implications

Current and anticipated advancements in the field of AI will play an influential role in decision-making related to adopting novel and innovative machine learning based techniques in health care. Clinical applications of AI include analysis of electronic health records, medical image processing, physician and hospital error reduction [107] AI applications in workflow optimization include payer claim processing, network coordination, staff management, training and education, supply costs and management [107] For example, the top three applications of greatest near-term value (based on the impact of application, likelihood of adoption and value to health economy) are reported to be robot-assisted surgery (valued at $40 B), virtual nursing assistants ($20B) and administrative workflow assistance ($18 B) [108]. Applications with lowest estimated potential value include preliminary diagnosis ($5B), automated image ($3B) and cyber-security ($2B) [108]. Our findings warrant the understanding of perspectives and beliefs of those adopting ANN-based solutions in clinical and non-clinical decision-making.

Patients and families are accessing health information in real-time with the array of AI or ANN based health care solutions available to them in an open and unstructured market. Clinical applications of ANN-based solutions can have implications on the changing role of health care providers as well team dynamics and patterns in workflow. The changing role of the physicians has been at the forefront of recent debates on AI, with some anticipating the positive impacts of augmenting clinical service with AI based technologies, e.g., enabling early diagnosis, or improving understanding of a patient’s medical history with genetic sequencing [109]. Literature suggests a need for bridging disciplines in order to enable of clinicians to benefit from rapid advancements in technology [101] In addition to the implications for clinical decision-making, interprofessional team dynamics and processes can be expected to change. For example, a US based hospital has collaborated with a game development company to create a virtual world in which surgeons are guided through scenarios in the operating room using rules, conditions and scripts to practice making decisions, team communication, and leadership [110].

As policy-makers adopt strategies towards a value-based, patient-centred model of care delivery, decision-makers are required to consider the readiness of health care organizations for successful implementation and wide-scale adoption of AI or ANN based decision-support tools. Factors such as easier integration with hospital workflows, patient-centric treatment plans leading to improved patient outcomes, elimination of unnecessary hospital procedures and reduced treatment costs can influence wider adoption of AI-based solutions in the health care industry [107]. Challenges in uptake include the current inability of AI-based solutions to read unstructured data, the perspectives of health care providers using AI-based solutions, and the lack of supportive infrastructure required for wide-scale implementation [107]. For improved organizational readiness, the governance and operating model of health care organizations need to enable a workforce and culture that will support the use of AI to enhance efficiency, quality and patient outcomes [108].

Machine learning from unstructured data (e.g. patient health records, photos, reviews, social media data from mobile applications and devices) remain a critical unmet need for hospitals [107, 111]. Currently, most of the data in health care is unstructured and difficult to share [107] Wide-scale implementation and adoption of AI service solutions requires strong partnerships between AI technology vendors and health care organizations [107]. Policies encouraging transparency and sharing of core datasets across public and private sectors can stimulate higher levels of innovation-oriented competition and research productivity [112].

### Theoretical implications

Several theoretical implications emerge from our study findings. Healthcare organizations are complex adaptive systems embedded in larger complex adaptive systems[113]; health care organizational decision-making can appropriately rely on ANN as an internalized rule set. The change of health care delivery from single to multiple settings and providers has led to new complexities around how health care delivery needs are being structured and managed (e.g., support required for delivering collaborative care or patient participatory medicine) [1]. Traditional decision-making processes based on stable and predictable systems are no longer relevant, due to the complex and emergent nature of contemporary health care delivery systems [1]. Yet the health care organizational decision-making literature suggests the focus of decision-making persistently remains on problems that are visible, while the larger system within which health care delivery organizations exist remains unacknowledged [1]. Using complex adaptive systems (CAS) theory to understand the functionality of AI can provide critical insights: first, AI enhances adaptability to change by strengthening communication among agents, which in turn fosters rapid collective response to change, and further, AI possesses the potential to generate a collective memory for social systems within an organization [114].

The theory of CAS has been used as an alternative approach to improve our understanding and scaling up of health services; CAS theory shifts decision-making towards embracing uncertainty, non-linear processes, varying context and emergent characteristics [115]. Interdependent organizational factors such as clinical practice, organization, information management research education and professional development, are built around multiple self-adjusting interacting systems [116]. Agents (e.g. users of the system) respond to their environment based on internalized rule sets that are not necessarily explicit, shared or need to be understood by another agent [116]. Although lacking the ability to explain decision-making, ANN-based decision-support tools enable health care organizational decision-makers to respond to complex and emergent environments using incoming and evolving data.

## Conclusion

Our study found artificial neural networks can be applied across all levels of health care organizational decision-making. Influenced by advancements in the field, decision-makers are taking advantage of hybrid models of neural networks in efforts to tailor solutions to a given problem. We found ANN-based solutions applied on the meso- and macro-level of decision-making suggesting the promise of its use in contexts involving complex, unstructured or limited information. Successful implementation and adoption may require an improved understanding of the ethical, societal, and economic implications of applying ANN in health care organizational decision-making.

## Supporting information

S1 Checklist Preferred Reporting Items for Systematic Reviews and Meta-Analyses (PRISMA) checklist. (PDF)

S1 Appendix Search strategy and syntax. (PDF)

S2 Appendix Summary of findings. (PDF)

S3 Appendix Glossary of terms. (PDF)

S1 Workflow Preferred Reporting Items for Systematic Reviews and Meta-Analyses (PRISMA) flowchart. (PDF)
