# Supplementary material for: Applications of artificial neural networks in health care organizational decision-making: A scoping review
Source: PLoS One. 2019 Feb 19;14(2):e0212356. doi: 10.1371/journal.pone.0212356 (PMC6380578; doi:10.1371/journal.pone.0212356)
Supplement: S1 Appendix — (PDF) [file pone.0212356.s002.pdf]

Discipline: Business and Management

Database	ABI/Inform Global
Search Syntax	(((artificial neural network) OR (neural net) OR (neural AND nets) OR (artificial neural networks)) AND ((health care organization) OR (health organization) OR (health care organization) OR (health care organisation) OR (health care AND management)) AND ((a decision making model) OR (decision AND support) OR decision)) AND peer(yes) AND (stype.exact("Scholarly Journals" OR "Conference Papers & Proceedings") AND la.exact("ENG"))
	((MAINSUBJECT.EXACT("Decision support systems") OR MAINSUBJECT.EXACT("Neural networks") OR MAINSUBJECT.EXACT("Expert systems") OR MAINSUBJECT.EXACT("Intelligence") OR MAINSUBJECT.EXACT("Fuzzy logic")) AND (MAINSUBJECT.EXACT("Health care") OR MAINSUBJECT.EXACT("Health care management") OR MAINSUBJECT.EXACT("Health care networks") OR MAINSUBJECT.EXACT("Health care delivery"))) AND peer(yes) AND stype.exact("Scholarly Journals" OR "Conference Papers & Proceedings")
Database	JSTOR
Search Syntax	(((((artificial neural network*) OR (machine learning)) OR (artificial intelligence)) AND (healthcare organi?ation)) AND (decision making))<br>Narrow by:<br>Item Type: Articles, Research Reports<br>Language: English<br>Subject: Biological Sciences, Business, Communication Studies, Developmental & Cell Biology, Economics, Education, Engineering, Finance, General Science, Health Policy, Health Sciences, History of Science, Labor & Employment Relations, Library Science, Management & Organizational Behavior, Mathematics, Political Science, Population Studies, Psychology, Public Health, Public Policy & Administration, Sociology, Statistics, Technology

Discipline: Computer Science

Database	Advanced Technologies and Aerospace Database
Search Syntax	2 + Limit: Scholarly Journals and Conference Papers/Proceedings<br>((MAINSUBJECT.EXACT("Decision support systems") OR MAINSUBJECT.EXACT("Neural networks") OR MAINSUBJECT.EXACT("Expert systems") OR MAINSUBJECT.EXACT("Intelligence") OR MAINSUBJECT.EXACT("Fuzzy logic")) AND (MAINSUBJECT.EXACT("Health care") OR MAINSUBJECT.EXACT("Health care management") OR

	MAINSUBJECT.EXACT("Health care networks") OR MAINSUBJECT.EXACT("Health care delivery"))) AND peer(yes) AND stype.exact("Scholarly Journals" OR "Conference Papers & Proceedings"))
Database	ACM Digital Library
Search Syntax	(+artificial +neural +network* health care healthcare decision making decision-making organization* organisation*)

Discipline: Health Administration

Database	Ovid Healthstar 1966 to November 2017
Search Syntax	
#	Searches
1	exp Algorithms/ or exp "Neural Networks (Computer)"/
2	exp Artificial Intelligence/ or exp Expert Systems/
3	exp Machine Learning/
4	1 or 2 or 3
5	exp Health Services Administration/ or exp "Delivery of Health Care"/ or exp Health Facility Administrators/
6	exp Decision Making, Organizational/
7	exp Decision Making/
8	6 or 7
9	4 and 5 and 8
42	limit 9 to english
Database	Ovid MEDLINE: Epub Ahead of Print, In-Process & Other Non-Indexed Citations, Ovid MEDLINE® Daily and Ovid MEDLINE® 1946-Present
Search Syntax	
#	Searches
1	exp Artificial Intelligence/ or exp Algorithms/ or exp "Neural Networks (Computer)"/
2	exp Machine Learning/
3	exp Fuzzy Logic/ or exp Expert Systems/
4	1 or 2
5	1 or 2 or 3
6	exp Hospital Administration/ or exp "Delivery of Health Care"/
7	(organization and management).mp. [mp=title, abstract, original title, name of substance word, subject heading word, keyword heading word, protocol supplementary concept word, rare disease supplementary concept word, unique identifier, synonyms]
8	exp Decision Making/
9	6 or 7 or 8
10	4 and 9
11	5 and 9

12	5 and 6
13	5 and 7
81	limit 13 to english language
