# Supplementary material for: Applications of artificial neural networks in health care organizational decision-making: A scoping review
Source: PLoS One. 2019 Feb 19;14(2):e0212356. doi: 10.1371/journal.pone.0212356 (PMC6380578; doi:10.1371/journal.pone.0212356)
Supplement: S2 Appendix — (PDF) [file pone.0212356.s003.pdf]

**Author**	**Year**	**Study Area**	**Study Aim**	**Objective for using ANN**	**Taxonomy used**	**Sample size (N), training/test size**	**Reported Accuracy Rate %**
Acharya, U. R. et al.	2013	Cardiovascular, Medical Imaging	Compare computer aided diagnositic (CAD) technqiues to effectively differentiate between symptomatic and asymptomatic patients	Classification	Radial basis probablistic neural network	N = 146 images<br>N = 346 images<br><br>Training: 2/3 of dataset<br><br>Testing: 1/3 of dataset	93.1% and 85.3%
Aguiar, F. S., et al.	2016	Infectious Disease	Develop and evaluate ANN models for classification and risk group assignment for pulmonary tubercolosis in hospitalized patients	Diagnosis	SOM, MLP	N = 315 cases	81%
Ahmed, S. S. et al.	2017	Chron's Disease	Classification of Chron's Disease medical images using neuro-fuzzy automated classification	Diagnosis	Hybrid: BPNN, Neurofuzzy model	N = 387 patients	97.67%

Aktaruzzaman, M. et al.	2015	Sleep	Improve the automatic distinction of sleep states using heart rate variability based features only	Classification	FFNN	N = 20 polysomnographic recordings	68.4% to 83.8% (REM vs NREM) 67.6% to 71.3% (WAKE vs SLEEP)
Amer, M. B. et al.	2011	Potassium Disturbances	Propose a neural fuzzy system for diagnosis of potassium disturbances	Diagnosis	Hybrid: Neuro-fuzzy	N = 63 subjects Training set: 63 Testing set: 16	87%
Anderson, I. et al.	2007	Physical Activity	Explore potential use of an unaugmented commodity technology (mobile phone) as a health promotion tool	Analysis	ANN	N = 9 participants	70%
Atoui, H., et al.	2008	Cardiovascular, Telemedicine	Present an overview of an intelligent portable ECG monitor	Decision-making	Hybrid: ANN, Fuzzy logic	N/A	N/A
Bajo, J., et al.	2009	Telemedicine, Organizational Behaviour: Case-based planning	Present an intelligent environment developed for monitoring patients case based planning in hospital environment	Decision-making	Hybrid: RTPW (routing problems with time windows) based on Kohonen	N = 200 cases	N/A

					networks		
Barriga, A. et al.	2016	Telemedicine, Rehabilitation, Fall Detection, Elderly	Propose a vision-based system as an automatic, non-instrusive solution for telerehabilitation and telecare for elderly people	Pattern recognition	ANN	N/A	N/A
Belmonte, M.	1997	Other: Autism; Medical Imaging (EEG)	Application of ANN to predict behavioural performance among autistic people from EEG analysis	Classification	FFNN	N = 399 autistic auditory, N= 365 autistic visual, N=96 normal auditory, N=105 normal visual	minimum 80%
Berardi, V. L., & Zhang, G. P.	1999	Medical Diagnosis, Finance	To investigate effects of unequal misclassification costs in the performance of neural network classifiers	Classification	ANN	N = 7,200 cases Training set: 75% Testing set: 25%	N/A

Boilot, P. et al.	2002	Infectious disease	Use an electronic nose to detect and classify bacteria in eye and ENT infections	Classification	MLP, Radial Basis Function (RBF)	N=180 readings; 6 bacteria x 3 dilutions x 10 readings (eye) N=129 readings (ENT)	97.3% (Eye bacteria) 97.6% (ENT)
Bourdès, V. et al.	2011	Cardiovascular, Pharmacology	Identify determinants of persistence of evidence-based cardiovascular medications	Prediction	MLP	N = 4850 patients<br><br>Training set: 70% Testing set: 30%	N/A
Buscema, P. M. et al.	2017	Organizational Behaviour: Healthcare Corruption	Analyse the phenomenon of corruption in the health sector across the world	Analysis	Other: Auto-contractive map	N = 107 observations	N/A
Cao, Q. et al.	2012	Finance	Compare accuracy of linear autoregressive moving average (ARMA) model and nonlinear neural network model in producing forecasts of medical cost inflation rates	Forecasting, Hypothesis testing	FFNN	N = 139 monthly observations	N/A

Carlucci, D. et al.	2013	Organizational Behaviour: Service quality and patient satisfaction	Understand key service quality dimensions from the outpatients point of view	Identify factors	ANN	N = 547 questionnaires	N/A
Cevenini, G. et al.	2008	Maternal-fetal Health, Medical Imaging	Estimate fetal weight using ANN with echobiometric parameters (i.e. ultrasound)	Prediction	FFNN	N=4075 fetuses<br>Training set: 1600<br>Validation set: 1600<br>Testing set: 875<br><br>System applied in clinical practice to 61 pregnant women	N/A
Chao, C. et al.	2012	Renal Disease	Construct prognosis model for dialysis-related conditions in the surgical ICU	Classification	ANN	N = 243 patients	86.26%
Chesney, T. et al.	2006	Trauma	Comparison of data analysis of trauma data using ANN and logistic regression	Prediction	ANN	N = 1,658 records	80%
Chong, A. et al.	2015	Organizational Behaviour: Health technology assessment/adoption	Exploring the role of unified theory of acceptance and use of technology, demographics & personality in adoption of radio	Prediction	BPNN	N = 252 physicians and nurses<br>Training set: 90%<br>Testing set: 10%	N/A

			frequency identification (RFID) technology in a clinical setting				
Delen, D. et al.	2012	Cardiovascular, Surgery	Illustrate predictive and explantory analysis of large data sets	Prediction	MLP	N = 60,000 admission records	87.74%
Emanet, N. et al.	2014	Pulmonary disease	Develop diagnostic tool for pulmonary diseases as a decision support tool using machine learning methods	Classification	MLP	N=40 patients, 20 diagnosed with asthma and 20 normal	80% (85% when FEV and FVC1 are added)
Ernest Preston, G. et al.	2002	Organizational behaviour: ICU resource management	Develop and compare a neural network model for predicting ICU survival to logistic regression model	Prediction	ANN	N = 2,200 cases	79.50%
Eswaran, C., & Logeswaran, R.	2012	Organizational Behaviour: Decision-making	Present a dual hybrid model for support of health care decision making	Forecasting	Hybrid: linear regression, neural network and fuzzy models	N/A	N/A
Goodey RD, et al.	2000	Referral, Oral Care	Evaluate three primary to secondary	Decision-Making	ANN	N = 107 referral decisions	75%

			care referral strategies				
Grossi E, et al.	2014	Cardiovascular, Clinical Psychology, Telemedicine	Provide a semantic map analysis of underlying connection scheme among several psychological variables within a single individual among HF patients	Pattern recognition, Identify relationships	Other: Auto Contractive Map	N = 9 patients	N/A
Gunasekaran M, et al.	2017	Cardiovascular	Diagnose heart disease	Diagnosis	Hybrid: ANFIS, multiple kernal learning (MKL)	N = 250 observations	N/A
Hanafizadeh P, et al.	2010	Organizational Behaviour: Employee motivation	Evaluate effect of motivation of hospital employees on patient satisfaction	Pattern recognition	ANN	N = 300 questionnaires<br><br>Training set: 70 questionnaires<br>Testing set: 30 questionnaires	N/A
Hassan Y, et al.	2002	Medical Diagnosis	Propose methodology for hybrid system (combining neural network systems with rough sets theory) used for	Preference, Classification	Hybrid: Rough neural networks	N= 178 patients<br>N= 246 patients<br><br>Training set: 50%<br>Testing set: 50%	N/A

			decision and classification support				
Houston AL, et al.	1999	Cancer, Information Systems	Propose an architecture usable by medical knowledge information systems that will permit data mining across several medical information sources	Clustering, Identify relationships	Hybrid: Hopfield and Kohonen neural network	N/A	N/A
Hsu S, et al.	2008	Finance	Propose alternative risk adjustment model	Risk Adjustment	Hybrid: SOM, BPNN	N = 5,557 individuals	N/A
Ibrahim F, et al.	2010	Infectious Disease	Diagnose and classify early risk in dengue patients using bioelectrical impedance analysis (BIA) and artificial neural network (ANN).	Diagnosis, Classification	MFNN	N = 430 patients<br><br>Training set: (303) 70%<br>Testing set: (127) 30%	96.27%
#VALUE!	2015	Cardiovascular, Data Mining	Study and compare single data mining techniques with hybrid to predict heart disease diagnosis and	Diagnosis	ANN	N/A	N/A

			formulate treatment plan				
Jamei, M. et al.	2017	Readmissions	Develop and pilot a predictive model to accurately identify high-risk patients	Prediction	ANN	N = 323,813 inpatient stays	95%
Karan O. et al.	2012	Diabetes	Diagnose diabetes as a case study	Classification	MLP, FFNN	Training set: 456<br>Testing set: N/A	N/A
Kudyba S, Gregorio T.	2010	Organizational Behaviour: Factors impacting patient outcomes	Identify factors within the patient treatment and outcomes process that produce high variances (e.g. excesses) in performance metrics.	Analysis	ANN	N= 43,000 cases	N/A
Kudyba S, et al.	2006	Finance, Chronic Disease	Illustrate predictive modelling techniques to identify sources of future high resource demand	Prediction	ANN	N = 54,206 health plan members	81% to 83%
Kulkarni P, et al.	2016	Readmissions	Compare statistical approaches for predicting the likelihood of readmission to hospital within 30 days of discharge	Prediction	ANN	Training set: 75%<br>Testing set: 25%	100%

Kupusinac A, et al.	2016	Cardiovascular	Promote cardiometabolic risk estimation that saves time and money	Risk Estimation	Hybrid: ANNs guided by evolutionary algorithm	N = 2191 patients	90%
Lamers SL, et al.	2008	Infectious Disease, Genotypic testing	Evaluate different strategies of feature selection and neural network optimization	Prediction	Hybrid: Evolved ANNs	N = 149 sequences	75.50%
Larder B, et al.	2008	Pharmacology, Infectious Disease	Development and application of ANN models as alternative tools for interpretation of HIV genotypic drug resistance data	Prediction , Model treatment response	ANN	N = 351patients (prediction)<br><br>N = 511 patients (treatment model)	87% for prediction 78% for modelling treatment response
Lee CW, Jung-A P.	2001	Infectious Disease	Provide correct classifications of AIDS versus HIV status patients	Classification	ANN	Training set: 1026 patterns<br><br>Test set: 145 patterns	64% to 65%
Lim CW, Kirikoshi T.	2008	Organizational Behaviour: Prescription behaviour	Build predictive models for understanding the relevance and limitations of promotional spending on prescription responsiveness	Analysis	Hybrid: Genetic algorithm-partial least squares (GAPLS) analysis with neural networks	N = 3 major brands with 11 data variables	N/A

Lin C. e al.	2014	Pain Management	Propose a decision-making framework for adaptive pain management	Approximation	ANN	N = 89 patients	N/A
Lin, W-B.	2013	Organizational Behaviour: Crisis Management	Investigate crisis management mechanisms in hospitals	Hypothesis testing	Hybrid: Nonlinear fuzzy	N = 159 data points	N/A
Lin W-B et al.	2016	Organizational Behaviour: Crisis Management	Discuss management variables influencing the mechanism of crisis management in public hospitals	Hypothesis testing	Hybrid: Nonlinear fuzzy	N = 159 data points	N/A
Liu N.T. et al.	2014	Trauma	Develop and validate a model for predicting the need for life saving interventions among trauma patients	Prediction	MLP	N = 79 patients	89.80%
Ma C-M. et al.	2012	Renal disease	Construct a prognosis model for predicting death and dialysis in ICUs	Prediction	MLP	N = 293 patients	Death dataset: 89.2% Dialysis dataset: 91.1%
Makantasis, K. et al.	2016	Fall Detection, Elderly	Propose a fall detection system that uses 2D image analysis and relationship between camera coordinate	Classification	Hybrid: FFNN and Island Genetic Algorithm	N =50 fall incidents (minimum)	90% to 97%

			system and real 3D space.				
Malliaris ME, Pappas M.	2011	Finance	Compare two models built to determine best indicators of revenue generation among nonprofit foundations supporting hospitals of different sizes	Model Revenue	ANN	N = 182 hospitals	99.80%
Marble RP, Healy JC.	1999	Trauma	Introduce the application of artificial neural networks to trauma complications assessment	Diagnosis	ANN	N = 912 trauma cases<br><br>Training set: 256<br>Testing set: 259	N/A
Mobley BA, et al.	2000	Cardiovascular	Produce a prototype of a neural network system that can detect patients who do not need coronary angiography	Prediction	ANN	N = 763 patients<br><br>Training: 332 records<br>Testing: 100 records	N/A
Okoroh MI, et al.	2006	Organizational Behaviour: Risk Management	Provide optimum solution and best value approach for managing healthcare non-clinical services	Risk Assessment	ANN	N = 60 managers<br><br>Testing set: 340 cases<br>Training set: 85	N/A

			and possible risky support service encounters			cases	
Okoroh, M. I. et al.	2007	Organizational Behaviour: Non-clinical health care management	Create a national healthcare service facilities risk exposure system allowing healthcare managers to provide an early warning signal to health care managers	Prediction	BPNN	N= 60 healthcare operators	N/A
Olatubosun O. et al.	2015	Diabetes	To diagnose diabetes	Diagnosis	Other: Maximum Covariance BPNN	N = 442 patients<br><br>Training set: 70%<br>Testing set: 15%<br>Validating set: 15%	90.9% to 100%
Oliva JT, Garcia Rosa JL.	2017	Epilepsy; Medical Imaging (EEG)	Verify if the choice of an epileptic EEG segment as reference can affect performance of classifiers built from data	Classification, Prediction	MLP, BPNN	N=200 EEG segments<br><br>(100 normal and 100 epileptic)	100%
Oztekin, A.	2012	Surgery (Transplantation)	Develop models to predict performance of thoracic	Prediction	ANN	N = 6,1391 records and 443 variables	78.15% to 82.24%

			transplants				
Pappada S.M. et al.	2013	Trauma, Surgery	Evaluate a model for glucose prediction in critically ill trauma and post-operative cardiothoracic surgical patients	Prediction	FFNN	Training set: 14 patients<br>Testing set: 5 patients<br><br>Training set: 19,989<br>Testing set: 9,405	53.6% for high glucose values<br>96.7% for normal glucose valyes
Pendharkar PC, Rodger JA.	2003	Organizational Behaviour: Factors impacting estimating resources required	Demonstrate using a data envelopment analysis (DEA) model to screen training cases and application of ANN to forecast number of employees in health care industry	Forecasting	BPNN	N = 175 hospitals<br><br>Training set: 100 hospitals<br>Testing set: 75 hospitals	54.7% to 100%
Pernek I, Ferscha A.	2017	Surgery	Review literature on different approaches for recognition of context in surgery	Classification	ANN	N/A	N/A
Pogorelc B, et al.	2012	Elderly	Propose a system for the early recognition of health problems manifesting in form of gait	Hypothesis testing	ANN	N = 9 subjects<br><br>Dataset: 141 recordings	95% to 100%

Pollettini J.T. et al.	2012	Organizational Behaviour: Pediatric health surveillance	Create a computer-aided automatic assignment of patient surveillance levels	Classification	MLP	N=100 medical records<br>Training set: 90%<br>Testing set: 10%	64.40%
Rangayyan, R. M., & Wu, Y. F.	2008	Osteoarthritis, Screening	Propose use of statistical parameters of vibroarthrographic signals	Classification	ANN	N = 89 VAG signals	82%
Samanta, B., & Nataraj, C.	2008	Cardiovascular	Compare soft computing techniques (GA-ANFISH and GA-ANN) for diagnosis of heart conditions	Classification	Hybrid: ANFIS, Genetic Algorithm-ANN (MLP)	N/A	100%
Sangi M. et al.	2015	Diabetes	Develop a risk advisor model to predict the chances of diabetes complications according to the changes in risk factors	Prediction	ANN	N/A	N/A

See CK, et al.	2010	Diabetes	Identify plantar pressure distribution in normal patients, type-2 diabetes with or without neuropathy	Classification	BPNN	N=85 patients	87.5% for diabetic patients without neuropathy<br><br>75% for diabetic patients with neuropathy
Shang J.S. et al.	2000	Infectious Disease	Derive and validate use of neural networks and logistic regression for diagnosis of community acquired Methicillin-resistant Staphylococcus aureus (MRSA) infections	Diagnosis	ANN	Training set: 354 patients<br>Testing set: 118 patients	89.83%
Sharma DK, Hota HS.	2013	Dermatology	Propose ensemble model for classification of different categories of Erythemato-Squamous diseases	Classification	Hybrid: ANN, SVM	N = 365 instances<br>Training set: 266 (76%)<br>Testing set: 99 (27%)	99.25% to 98.99%

Sheikhtaheri A. et al.	2014	Organizational Behaviour: Clinical decision-making; Review	Review challenges associated with the use of ANN in clinical decision-making	Classification	ANN	Various	90-100%
Singh K et al.	2016	Telemedicine	Integrate artificial neural intelligence in domain of healthcare monitoring	Decision-making	Hybrid: ANFIS	N/A	N/A
Süt N, Senocak M.	2007	Cardiovascular	Compare predictive accuracies of diagnosis of coronary artery disease using different types of artificial neural network and statistical methods	Diagnosis	MLP, RNN	N = 1803 cases	79% to 83.9% (MLPNN)<br><br>84.7% (RNN)
Veredas F.J. et al.	2009	Trauma	Use a hybrid learning approach to design a computational system for automatic tissue identification on wound images	Classification	Hybrid: Bayesian networks, k neural networks, MLP	N = 113 photographs	N/A
Verma B, Hassan SZ.	2011	Information Systems	Present a hybrid ensemble approach for classification in medical databases	Classification	Hybrid: MLP, SOM, K-Means	N = 768 cases<br><br>Training set: 200<br>Testing set: 100	89% to 100%

Vivekanandan T, Iyengar NCSN.	2017	Cardiovascular	Find optimal subset of attributes from complete attribute set for diagnosing heart disease	Prediction	Hybrid: Fuzzy AHP with FFNN	N = 300	83% to 85%
Vu T.H.N. et al.	2010	Cardiovascular, Telemedicine	Propose a neural network to recognize Heart Rate Variability (HRV) patterns related to coronory heart disease (CHD) risk in consideration of daily activities	Pattern recognition	Hybrid: Poincare-based HRV patterns discovering Incremental Artificial Neural Network, ANN	N = 11 scenarios<br><br>Each scenario gives dataset of 5400 samples	50% to 95%
Waghulde NP, Patil NP.	2014	Cardiovascular	Evaluate application of neural network in predicting presence of heart disease	Prediction	Hybrid: Genetic Neural, BPNN, FFNN	N = 50	84% to 98%
Wu H-J. et al.	2009	Information Systems	Use artificial neural network as part of location scheme that can learn geography features and adapt to the real world	Analysis	BPNN	Training set: 60 sets<br>Trresting set: N/A	60% to 90%

Xu M. et al.	2013	Organizational Behaviour: ED patient arrivals	Provide a comprehensive comparison of different computational methods to model patient arrivals	Prediction	ANN	Training set: 328 days<br>Testing set: 37 days<br><br>Training set: 90%<br>Testing set: 10%	N/A
Zear A. et al.	2018	Information Systems, Telemedicine	Propose an algorithm for multiple digital watermarking for healthcare applications in order to prevent medical patient identify theft.	Optimization	BPNN	N/A	N/A
Zhang S. et al.	2009	Obesity, Data Mining	Compare logistic regression with six data mining techniques to improve accuracy of prediction	Prediction	ANN	N = 16,523 instances	N/A
