# Supplementary material for: Applications of artificial neural networks in health care organizational decision-making: A scoping review
Source: PLoS One. 2019 Feb 19;14(2):e0212356. doi: 10.1371/journal.pone.0212356 (PMC6380578; doi:10.1371/journal.pone.0212356)
Supplement: S3 Appendix — (PDF) [file pone.0212356.s004.pdf]

Glossary of Terms

Artificial Intelligence: The science and engineering of making intelligent machines (computer programs), where intelligence is the computational part of the ability to achieve goals (1).

Artificial Neural Networks: A statistical model comprised of interconnected processing elements configured through iterative exposure to data (2).

Attribute: Machine learning term(3),
Synonym: *feature*.

Backpropagation: Backpropagation refers to how the network is trained, i.e. involving a forward pass of the data processing an input vector, updating the connection weights and produce an output. This is followed by a backward pass at the output layer involving a pass back of the error term, i.e. the difference between the actual and desired output as a partial derivative of the transfer function (2).

Bayes Decision Rule: The function that assigns to each feature vector a label in a way that minimizes the probability of classification error (error rate)(3).

Bayesian Statistics: Statistical framework combining data with subjective prior information about parameter values in order to derive posterior probabilities of different models or parameter values(4).

Categorical Variable: A variable having only certain possible values without logical ordering of values, also referred to as nominal, discrete categorical variable or factor (5).

Causal Relationship: An established relationship showing an independent variable causing a change in a dependent variable. It also establishes how much of a change is shown in the dependent variable (6).

Classification: Ordering of related information into categories, groups or systems according to characteristics or attributes (6). A classification function maps from χ to γ, assigning a label to each feature vector (3).

Cluster Analysis: A method of statistical analysis where data sharing common traits or characteristics are grouped together (6).

Continuous Variable: A variable that can take a number of possible values (e.g. variable with at least 10 values) and can be plotted on a scatterplot. Certain meaningful calculations can be made using that variable (5).

Data Mining: The process of analyzing data to discover patterns and/or systematic relationships among variables (6).

Error rate: The probability that a classifier incorrectly labels an observation χ (3).

Feature: A numeric or categorical quantity used as input to a classifier (e.g. the length of an object)(3).
Synonym: *attribute*.

Feed-forward Networks: Artificial neural networks involving unidirectional flow of information without any feedback, i.e. from input layer, through hidden layers to the output layer (e.g. single-layer perceptron, radial basis function networks) (7).

Generalized linear Model: Statistical models that assume errors from the exponential family; predicted values are determined by discrete and continuous predictor variables and by the link function (4). A broad class of models made of three components: random (probability distribution of the response variable), systematic (explanatory variables), and link function (link between random and systematic functions) and include models such as linear regression, ANOVA, Poisson regression, logistic regression etc. (8).

Hybrid Models: A model combining or integrating multiple techniques (e.g. supervised and unsupervised learning) in a two stages: first by a clustering or classification technique in order to filter out unrepresentative training data, followed by constructing a prediction model (9, 10).

Hypothesis: A tentative explanation based on theory to predict a causal relationship between variables (6).

Linear regression model: Also called 'ordinary least squares' or 'general linear model', referring to regression for a continuous dependent variable and usually to the case where residuals are assumed to be Gaussian. The linear regression model is not to be confused with 'generalized linear model' where the distribution can take on several non-Gaussian forms (5).

Logistic regression model: A multivariable regression model relating one or more predictor variables to the probabilities of various outcomes. The most common is the binary logistic model which predicts the probability of an event as a function of several variables (11).

Machine Learning: A sub-discipline of artificial intelligence and a form of data analysis involving computers to continuously learn from data in order to perform tasks ranging from natural language processing to predicting outcomes. A key aspect being that as models are exposed to new information, they can adapt and produce reliable and consistent output(12).

Modelling: The creation of a physical or computer analogy to understand a given phenomenon, helping estimate the relative magnitude of the different factors involved (6).

Models: Representations of processes, principles or ideas often used for imitation or emulation (6).

Nonparametric testing: A test that makes minimal assumptions about the distribution of the data or about certain parameters of a statistical model. Nonparametric tests for ordinal or continuous variables are typically based on the ranks of the data values. Examples of nonparametric tests are the 2-sample Wilcoxon-Mann-Whitnehy test, the 1-sample Wilcoxon signed-rank test, the Spearman or Kendall tests (5).

Parametric test: A test which makes specific assumptions about the distribution of the data or parameters of a model. Examples include t-test (5).

Recurrent Feedback Networks: Artificial neural networks involving feedback loop in flow of information (e.g. Kohonen's self-organizing maps, Hopfield networks) (7, 13).

Statistical Analysis: Application of statistical processes and theory to the compilation, presentation, discussion and interpretation of numerical data (6).

Test Set: A collection $(\chi_1,\gamma_1),\ldots,(\chi_m,\gamma_m)$, used to validate the performance of a classifier(3).

Training Set: A collection $(\chi_1,\gamma_1),\ldots,(\chi_m,\gamma_m)$, where the (χ_i,γ_i) are labeled examples used for training(3).

References

1. McCarthy J. What is Artificial Intelligence? [Article]. In press 2007.
2. Scarborough D, Somers MJ. Neural Networks in Organizational Research: Applying Pattern Recognition to the Analysis of Organizational Behaviour. Washington, D.C.: American Psychological Association; 2006.
3. Columbia University. Glossary.
4. Bolker B, Brooks ME, Clark CJ, Geange SW, Poulsen JR, H SMH, et al. Generalized linear mixed models: a practical guide for ecology and evolution. Elsevier. 2008.
5. Vanderbilt University School of Medicine. Glossary of Statistical Terms. 2017.
6. University of Southern California. Glossary of Research Terms 2018 [Available from: http://libguides.usc.edu/writingguide/researchglossary.
7. da Silva I, Spatti H, Flauzino A. Artificial Neural Network Architectures and Training Processes. Artificial Neural Networks: A Practical Course. Switzerland: Springer International Publishing; 2017.
8. The Pennsylvania State University. 6,1 - Introduction to Generalized Linear Models: The Pennsylvania State University,; 2018 [Available from: https://onlinecourses.science.psu.edu/stat504/node/216/.
9. Tsai C-F, Hu Y-H, Hung C-S, Hsu Y-F. A comparitive study of hybrid machine learning techniques techniques for customer lifetime value prediction. Kybernetes. 2013;42(3):357-70.
10. Benvenuto F, Piana M, Campi C, Massone AM. A Hybrid Supervised/Unsupervised Machine Learning Approach to Solar Flare Prediction. The Astrophysical Journal. 2018:853:90.
11. Zhao DX, Leacche M, Balaguer JM, Boudoulas KD, Damp JA, Greelish JP, et al. Routine intraoperative completion angiography after coronary artery bypass grafting and 1-stop

hybrid revascularization results from a fully integrated hybrid catheterization laboratory/operating room. J Am Coll Cardiol. 2009;53(3):232-41.
12. Manghani A. A Primer on Machine Learning.
13. Sharma A, Chopra A. Artificial Neural Networks: Applications in Management. Journal of Business and Management. 2013;12(5):32-40.
