# Supplementary material for: Applications of artificial neural networks in health care organizational decision-making: A scoping review
Source: PLoS One. 2019 Feb 19;14(2):e0212356. doi: 10.1371/journal.pone.0212356 (PMC6380578; doi:10.1371/journal.pone.0212356)
Supplement: S1 Workflow — (PDF) [file pone.0212356.s005.pdf]

PRISMA 2009 Flow Diagram

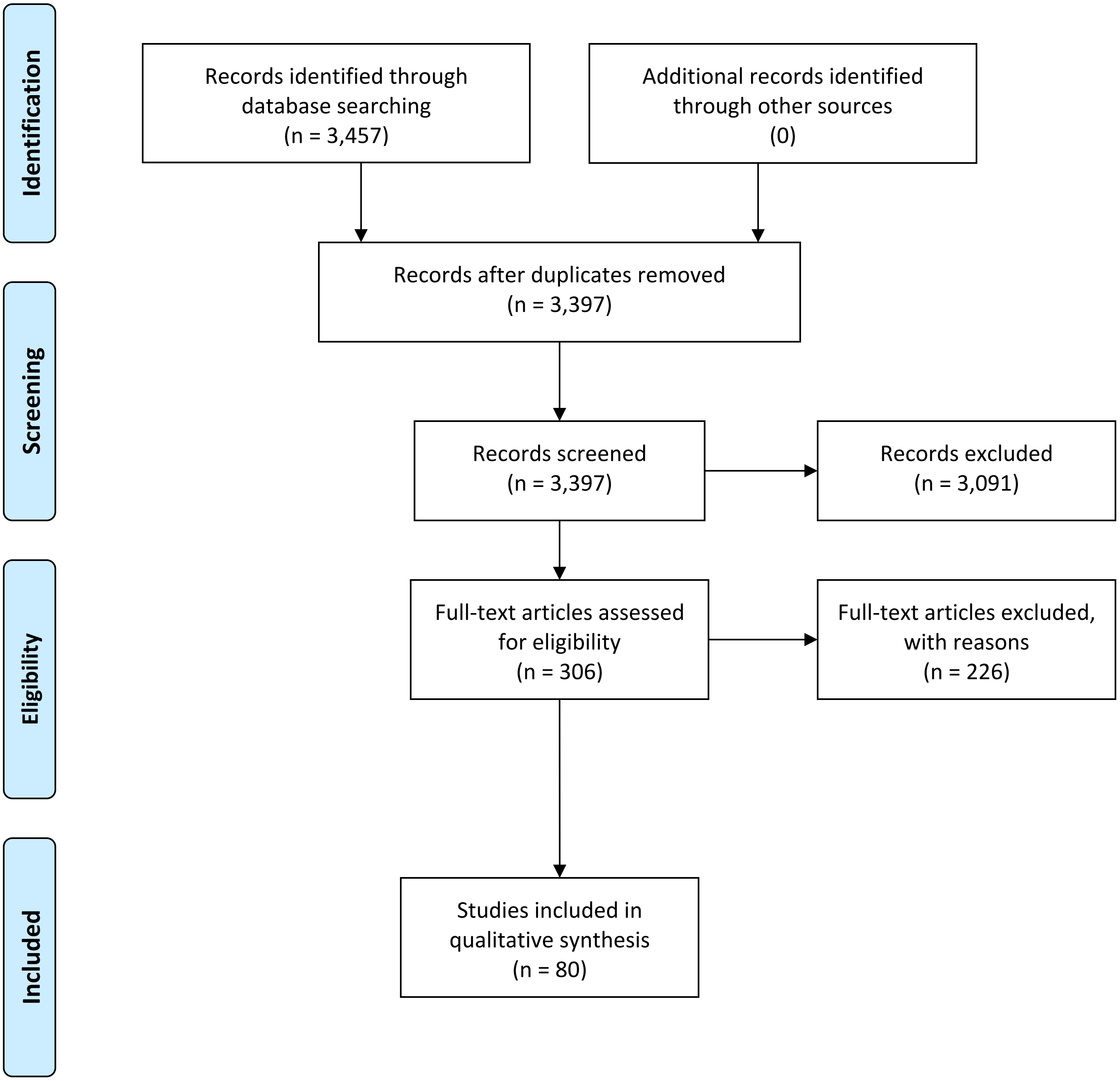

From: Moher D, Liberati A, Tetzlaff J, Altman DG, The PRISMA Group (2009). *P*referred *R*eporting *I*tems for *S*ystematic Reviews and *M*eta-*A*nalyses: The PRISMA Statement. PLoS Med 6(7): e1000097. doi:10.1371/journal.pmed1000097

For more information, visit www.prisma-statement.org.
